# Inhalation of Microplastics—A Toxicological Complexity

**DOI:** 10.3390/toxics12050358

**Published:** 2024-05-11

**Authors:** Myriam Borgatta, Florian Breider

**Affiliations:** 1 Center for Primary Care and Public Health (Unisanté-Lausanne), University of Lausanne, 1015 Lausanne, Switzerland; 2 Central Environmental Laboratory, Ecole Polytechnique Fédérale de Lausanne (EPFL), 1015 Lausanne, Switzerland; florian.breider@epfl.ch

**Keywords:** microplastics, inhalation, lung, human, toxicity

## Abstract

Humans are chronically exposed to airborne microplastics (MPs) by inhalation. Various types of polymer particles have been detected in lung samples, which could pose a threat to human health. Inhalation toxicological studies are crucial for assessing the effects of airborne MPs and for exposure-reduction measures. This communication paper addresses important health concerns related to MPs, taking into consideration three levels of complexity, i.e., the particles themselves, the additives present in the plastics, and the exogenous substances adsorbed onto them. This approach aims to obtain a comprehensive toxicological profile of deposited MPs in the lungs, encompassing local and systemic effects. The physicochemical characteristics of MPs may play a pivotal role in lung toxicity. Although evidence suggests toxic effects of MPs in animal and cell models, no established causal link with pulmonary or systemic diseases in humans has been established. The transfer of MPs and associated chemicals from the lungs into the bloodstream and/or pulmonary circulation remains to be confirmed in humans. Understanding the toxicity of MPs requires a multidisciplinary investigation using a One Health approach.

## 1. Introduction

The presence and toxicity of plastic in the environment have been studied for decades now, and plastic is recognized as a persistent organic pollutant that causes harmful health effects on living organisms [1]. Nevertheless, studies on human exposure to and the toxicity of plastic are scarce and focused predominantly on the oral route within contaminated food and beverages. Recent publications have reported the presence of plastic fragments in the human lungs, revealing inhalation as an additional route of absorption [2,3,4,5,6]. Plastic is a generic term including numerous polymers and rubbers that are used in the production of countless single-use and reusable products intended for human activities, such as toys, furniture, tires, vehicle interiors, medical and sport equipment, and carpets [7,8,9]. During the manufacturing process of these products, a plethora of chemicals, such as plasticizers, solvents, antioxidants, biocides, colorants, fillers, flame retardants, light stabilizers, nucleating agents, and fragrances, can be incorporated into the polymer matrix [10]. These additives impart additional properties to the polymers, such as functionality, longevity, color, brightness, and homogenous blending of the final product [11,12]. Plastics may also contain non-intentionally added chemicals, like by-products and breakdown products [13]. Therefore, one plastic item is not a composite blend of a polymer but a cocktail of different chemicals. With use and aging, plastic items wear out, releasing countless fragments into the soil, water, and the atmosphere [14,15]. MPs are plastic fragments with a size ranging from 5 mm to 0.001 mm, the smallest (<0.001 mm) being nanoplastics [16,17]. MPs are predominant as dust [18,19,20] and airborne particles [16,20,21,22,23] in urban areas [24] and indoor environments [23,25,26]. Dust is easily resuspended in the air with the action of air flow (i.e., wind, ventilation systems, passing vehicles, doors opening/closing), making MPs available for inhalation [8,27,28]. Human inhalation exposure to airborne MPs may vary during the day, depending on human activity and location. Workers may be exposed to indoor and outdoor airborne MPs during specific activities such as the production of plastic items, the treatment of plastic waste, road maintenance, and 3D printing [29,30,31,32,33,34,35,36]. Children crawling on the floor can lead to resuspending MPs in the air at short distance to their nose and mouth. Knowing that children and adults spend on average 19 h and 21 h per day indoors (i.e., at home, in the workplace, and in a vehicle) [37], respectively, inhalation can therefore be considered as a chronic exposure route to airborne MPs, with concentration peaks depending on the activities.

Little is known about the inhalation exposure of humans to MPs and the characteristics of these particles in the lungs. The interactions of MPs with the pulmonary environment (i.e., local effects) and their potential distribution throughout the human body need to be investigated. In the environmental field, studies have predominantly focused on relatively large MPs using analytical methods reliable for water, soil, and air samples. Analytical method improvements such as Raman and infrared microscopy (e.g., µFTIR), and pyrolysis–gas chromatography–mass spectrometry (Py-GC/MS) can nowadays provide detailed descriptions of the MPs present in human fluids and tissues [2,3,4]. Airborne MPs display a range of sizes, shapes, and chemical compositions that may influence both their lung deposition and pulmonary (local) and systemic toxicity [7,38,39]. Systemic effects occur when a chemical enters the lymphatic or circulatory system, which is interconnected with various organs and tissues [40]. Moreover, the hydrophobic surface of MPs likely promotes the adsorption of exogenous pollutants with high octanol–water partition coefficients (K_ow_), such polycyclic aromatic hydrocarbons (PAHs), organochlorinated biphenyls (PCBs), per- and poly-fluoroalkyl substances (PFASs), pesticides, and pharmaceuticals [7,41,42,43]. These adsorbed pollutants may be released in the lungs depending on the local conditions (e.g., temperature, pH, humidity, macrophages, residence time). MPs can thus serve as reservoirs for additives and as carriers for hazardous pollutants that may threaten human health [44,45,46,47].

**Figure 1:**
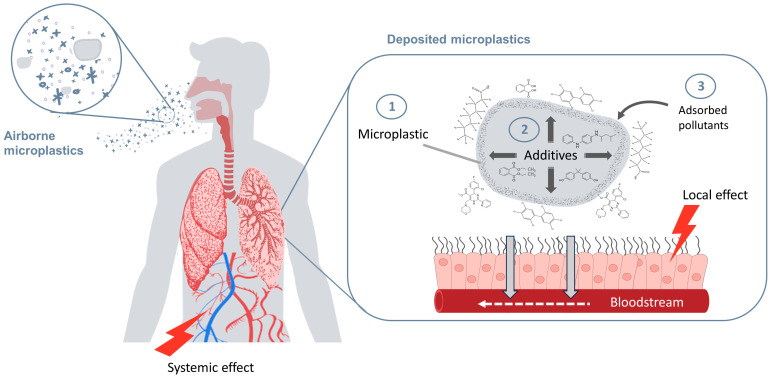
Inhalation exposure to microplastics (MPs) of varying physical and chemical properties. Three situations related to MP deposition in the pulmonary system may harm human health by acting simultaneously or independently. 1. MP particles deposited in the lungs. 2. MPs as reservoirs of additives. 3. MPs as carriers of environmental pollutants. This figure was created with https://www.canva.com/ (Accessed on 15 March 2024).

The toxicological assessment of airborne MPs via inhalation relies on a combination of physical and chemical factors that could potentially act simultaneously to induce local and/or systemic toxic effects (Figure 1). The aim of this communication is to outline three levels of complexity that are important to consider when assessing inhalation exposure to MPs and human health: (1) MPs as deposited particles in the lungs, (2) MPs as reservoirs of additives, and (3) MPs as carriers of adsorbed pollutants. From our perspective, adopting a holistic approach facilitates a comprehensive assessment of the risks associated with MP exposure, thereby providing valuable insights for decision-makers in the public health and environmental protection domains.

### 1.1. MPs Deposited in the Lungs

As with other airborne particles, MPs with a size range of 1–5 µm are likely deposited in the nasopharyngeal and bronchial sections of the respiratory tract [48]. MPs of 1 µm and nanoplastics can reach the deepest lung regions (i.e., the alveoli), where vital gas exchanges with blood occur. Inhalation exposure to MPs can lead to an accumulation of 26 to 130 MPs per day in the human lungs [22]. Deposition of MPs in the respiratory system depends on their size, shape, and surface properties. Until now, only seven studies have characterized MPs in human lung tissues and bronchoalveolar lung fluid (BALF) using optimized analytical methods [2,3,4,5,6,49,50]. The results of these studies are summarized in Table 1. Among the polymers that were targeted in lung tissues, the predominant one were polypropylene (PP) and polyethylene terephthalate (PET) [2,4,5,49]. Focusing specifically on MP fibers, Chen et al., in 2022 [4], observed that the quantity of these fibers was twice as high in lung tumors than in normal tissues. Three other studies also primarily focused on fibers but in human BALF samples [3,6,50]. The MP size ranged from <5 μm to 34 μm, and the four dominant polymers were PE, PP, PET, and PS. A single study reported a concentration of 9.18 ± 2.45 MP fibers/100 mL of BALF [3]. In the BALF of children, up to 332 MP particles were found, and the concentration was even higher than in adult samples [50]. Within these studies, fibers, fragments, and films were detected. The length range reported was from <5.5 µm up to 2475 μm, with fibers usually exhibiting the most elongated shape. One study characterized the surface of the fibers as having rough and porous features with cracks or being significantly damaged [6]. These seven studies provide the first insights into the presence of MPs in human lungs. The detection and characterization methods used in these studies relied on infrared spectro-imaging (i.e., µFTIR, µRaman, LDIR). While these imaging techniques provide particle-based concentrations, their detection is limited by the spatial resolution of the instrument, typically ranging between 5–20 μm. This limitation hinders the detection of small MPs and nanoplastics. The identification of particles using these techniques is heavily influenced by particle aging, as most spectral libraries only contain data on virgin polymers unaffected by solar radiation, atmospheric gases, abrasion, and heat. Additionally, these techniques solely provide information on the nature of the polymer but not the additives present in the particles. Therefore, additives cannot be analyzed using these techniques. To our knowledge, Py-GC/MS has not been employed for analyzing MPs in human lungs up to now, although Py-GC/MS is not based on the spectral characteristics of the MPs but on the analysis of molecular tracers by mass spectrometry. This approach offers several advantages over infrared-based methods, since the detection of MPs is not limited by the size but by the mass. Py-GC/MS can provide information on the additives present in MPs. Detection and characterization methods based on infrared spectroscopy and mass spectrometry complement each other. Combining these approaches could enhance our understanding of the chemical and physical complexities of MPs, leading to a more comprehensive assessment of the nature of airborne MPs and, therefore, the potential health effects.

### 1.2. Toxicity of MPs in the Lungs

Alveolar macrophages are efficient in clearing particles with diameters >1 μm, while smaller particles tend to persist for longer period [51]. In the bronchial region, a layer of mucus lines the airway walls, where cilia actively vibrate to remove deposited particles back up to the esophagus within 24 h following particle inhalation [52]. In vitro studies showed that polystyrene (PS) microspheres of a 1 µm diameter can be internalized in less than 24 h by human alveolar A549 cells, altering the proliferation, metabolism, morphology, and cohesion of these cells [53,54,55]. The size and shape of polyethylene (PE) and PS MPs are related to cytotoxic effects in human cerebral and lung epithelial cells [56,57]. In addition to the size, the surface characteristics may induce pulmonary effects in animal-models exposed to PS microspheres by tracheal instillation [58]. Except in respiratory therapy, where spherical MPs are used as carriers for drug delivery application [59], humans are mainly exposed to airborne MPs with irregular shapes [6,49,50]. Workers exposed to polypropylene (PP) microfibers with an irregular shape and size coupled with lamellar fragmentation at the surface exhibited a 3.6-fold higher risk of respiratory symptoms compared to non-exposed individuals [60]. Toxicological studies on the effects of PP and other polymers with an irregular shape at the pulmonary level are lacking [61]. PS microspheres have been the most extensively studied polymer and shape in vitro and in vivo, demonstrating apoptosis in human alveolar macrophages, alveolar epithelial cells, and lung cancer cells. Alveolar lesions were reported in rodents exposed by inhalation to nylon dust and acrylic fibers [62]. Lung biopsies revealing acute alveolar injury and bronchiolitis were also documented in workers suffering from occupational interstitial lung disease (flock worker’s lung) after repeated and prolonged exposures to PE and nylon microfibers [29,63,64,65]. Chronic occupational exposure to PVC dust was associated with breathlessness symptoms and abnormal pulmonary function responses [31,60]. PVC and vinyl chloride were linked to lung inflammation and diseases, including cancers [66]. While evidence supports the effects of MPs on pulmonary cells in vitro and in animal models as well as on respiratory diseases in plastic industry workers, a causal link has never been established in humans. One reason for the lack of causality may be attributed to advances in methods specifically designed to characterize and quantify MPs, which are only recent. These methods hold promise for demonstrating a causal link in humans and, thus, a better understanding of the health risks of inhaled MPs.

Py-GC/MS detected PET, PE, and styrene polymers with sizes equal to or exceeding 0.7 µm in human blood samples [67], raising questions about the route and mechanism of absorption of MPs. Diffusion through the alveolar wall into the lymphatic system was reported for particles ranging between 0.1 and 1 mm in diameter [68]. After oral exposure, nano- and micro-plastics (polystyrene) penetrated the lymphatic and/or blood system in rodents [69,70]. PS microparticle absorption across membranes was reported to be fast, within minutes in the small intestine of rats orally exposed to PS microspheres [71,72]. PS MPs with a diameter ranging from 500 nm to 5 µm were detected in the blood and further distributed to the brain and the heart, with the highest concentrations found in the liver, kidneys, and lungs. In humans, the likelihood that MPs reach the placenta and the blood–brain barrier is starting to be tackled, along with their distribution into the liver, muscles, and brain [7]. In an in vitro 3D lung cell model, Rothen-Rutishauser et al. [54] showed that 1 μm PS particles either adhered to or were internalized in macrophages present on the epithelial surface. These immune cells aim to remove foreign particles through the bloodstream and the lymphatic system. Subsequently, MPs may also be found in the regional lymph nodes [73]. The relative contribution of lymphatic transport and mucociliary clearance varies among species [74] and is unknown with MPs in humans, nor are the systemic effects of MPs. A broad spectrum of MP toxic effects at the whole-organism level were observed in various species, mainly from marine ecosystems [75,76,77,78,79]. These effects span multiple life stages, encompassing developmental, behavioral, genotoxic, and metabolic aspects, along with increased mortality, immune responses, and organs dysfunction. To understand the potential local and systemic effects in humans chronically exposed to airborne MPs, the characterization and quantification of both airborne MPs in indoor environments and deposited MPs in the lungs would serve as a starting point. Basic toxicological principles assert that the frequency and dose (i.e., the mass of MPs deposited in the lungs) of exposure, as well as the residence time of MPs in the lungs, can determine the toxicity of both the MPs and the released chemicals (i.e., additives and adsorbed pollutants).

### 1.3. MPs as Reservoirs of Chemicals

MPs provide a durable reservoir for both classified and unclassified hazardous chemical intentionally or unintentionally incorporated in the polymer, which may leak into the pulmonary environment (i.e., lung tissues and fluids). Approximately 10,550 chemicals were identified as likely being used in plastics across various industrial sectors, and 4300 chemicals are likely used in plastic packaging [10,13]. Among these additives, up to half of them lack hazard classifications, with the others having been reported as having endocrine-disrupting effects, neurotoxicity, or reprotoxicity, such as phthalates, bisphenol A (BPA), triclosan, bisphenone, organotins, and brominated flame retardants [80]. Phthalates, used as plasticizers to impart flexibility to polymer plastics, are an example of additives that are not chemically bound to the polymer matrix and that can migrate to the surface of the product [81]. Phthalates are additives known to leak from plastic medical devices and accessories when in contact with solvents such as *n*-hexane and a mixture of ethanol/water in laboratory conditions [82,83]. Phthalates (along with other additives) are prevalent in indoor air and dust, particularly in environments with plastic products and devices such as hospitals and freshly decorated spaces [84,85]. After inhalation exposure, these additives have the potential to traverse the human alveolar–capillary membrane and be distributed throughout the body [86]. The lungs are a large surface of chemical absorption into the blood. This route of absorption bypasses the first-pass metabolism, a primary important “detoxifying” step inherent in the oral route [40]. Therefore, hazardous chemicals can be distributed to organs prior to being detoxified. To the best of our knowledge, no study has specifically investigated the inhalation of MPs containing additives nor the release of these chemicals in vivo or in a simulated pulmonary environment (e.g., pulmonary fluid, T°, pH, and enzymes). It is known that additives can be released by tire particles in the gastric and intestinal fluid [87], and similar studies should be performed with MPs exposed to simulated pulmonary fluid. Toxicological data are needed to understand the diffusion kinetics of plastic additives, their concentrations in the lungs and residence time, and their transfer across membranes. These data are important in assessing the potential effects of additives in the lungs and their absorption rate into the blood following inhalation exposure to MPs.

### 1.4. Toxicity of Additives

Given the multitude of additives and unintentionally added chemicals that could be present in MPs, we have chosen to summarize some effects related to phthalates and bisphenols as examples. In the lungs, phthalates interfere with the nuclear hormone receptor superfamily, influencing gene transcription [88] and lung maturation [89]. Following inhalation exposure, phthalates alter lung weight, cell proliferation, and the structure of the alveoli, potentially impacting gas exchange in rats [90,91,92]. Phthalates are associated with lung inflammation, oxidative stress, and a clinical decrease in the pulmonary functions [93,94,95,96,97,98,99]. Multiple studies have linked phthalates exposure with asthma and allergic reactions [100,101,102], including in children [97]. Oral exposure to phthalates also induces numerous lung effects, including abnormal lung histology [90], morphological changes in pneumocytes cells, and an increase in the number of alveolar macrophages in the pulmonary blood vessels [103]. These results demonstrate the affinity of phthalates for lung tissues, even when the exposure is not via inhalation. Bisphenols are other common plastic additives that cause pulmonary damage when in contact with the respiratory system. Similarly to phthalates, bisphenol A is associated with asthma [93,97,104,105,106]. Bisphenols also cause collapsed alveoli and morphological changes in the mucosa, blood vessels, and interalveolar septum [107,108], as well as lung development disorders [109,110]. These two examples of additives exhibiting lung toxicity are concerning considering the numerous other chemicals that may also leak into the lungs following MP inhalation exposure. This mixture of chemicals may induce individual, additive, or synergistic effects at both the local and systemic levels.

Phthalates and bisphenols (A and B) are endocrine-disrupting chemicals (EDCs) [111,112] with toxic effects on female and male reproduction, development, and various organs, along with the initiation of cancers [102,113,114,115,116,117,118]. In humans, toxic effects have been associated with phthalates such as retarded male reproductive development [119,120], altered semen quality [121,122], and allergic symptoms in children [97,104]. However, it remains unknown whether plastic additives such as phthalates and bisphenols can be absorbed in the bloodstream after MP inhalation exposure. Some evidence suggests that inhaled phthalates can cross the alveolar–blood membrane before being eliminated through urine [86,123,124]. Therefore, chronic exposure to MPs may be seen as a hidden health threat with significant toxic effects in humans [125].

### 1.5. MPs as Carriers for Environmental Pollutants

MPs can be a carrier for hazardous chemicals present in the environment [126,127]. MP surfaces are mainly hydrophobic and often have large specific surface areas favoring the adsorption of environmental pollutants (i.e., exogenous chemicals) with medium-to-high log K_ow_ constants, such as PAHs, PCBs, PFASs, and PBDEs [7,41,42]. PFASs, with log K_ow_ values ranging from 3 to over 6, are hydrophobic compounds that easily adsorb on plastics and are mainly associated with particulate matter. In the lungs, MPs and the carried hazardous pollutants come into contact with the epithelial fluid that lines the airways, extending from the larger airways down to the alveoli. This aqueous layer of biological solutes lining the pulmonary epithelium contains surfactants [128]. Composed mainly of phospholipids and proteins, the surfactant reduces surface tension, thus maintaining the alveoli in an open conformation optimal for gas exchange. With their amphiphilic structure, the phospholipids may favor the desorption and solubilization of hydrophobic chemicals. The local conditions (e.g., temperature, pH, humidity, enzymes) in the lungs may also influence the release of adsorbed pollutants. These chemicals become bioavailable in the surfactant and epithelial fluid, acting possibly similarly to the additives described previously. However, studies having specifically assessed the inhalation of MPs containing hazardous pollutants are still lacking. Studies performed with pulmonary fluids exist for particle matter (PM2.5 and PM10) and adsorbed metals and inorganic and organic matter such as PAHs [129]. MPs have been demonstrated to adsorb PAHs [130], but the desorption kinetics is unknown. In the lungs, PAHs easily diffuse through cell membranes and have been quantified in cancer tissues [131,132]. PAHs are reported to alter pulmonary functions and to be responsible for lung cancer [130]. PAHs have mutagenic, carcinogenic, and endocrine-disrupting effects, mainly due to enzymatic transformation leading to the formation of toxic metabolites [131]. After inhalation exposure, PAHs induced cancers, cardiovascular diseases [133], and blood hemolysis [134]. PAHs are an example of exogenous chemicals, among others, that MPs can carry. Therefore, airborne MPs can act as a “Trojan Horse”, hiding and carrying toxic exogenous and indigenous (additives) chemicals along the airways and likely throughout the entire organism.

## 2. Key Messages

In contrast to previous beliefs, MPs should no longer be considered inert and harmless particles. The physical heterogeneity of MPs can induce localized effects on the lungs. Evidence shows the pulmonary and systemic effects of MPs in animal or cell models, with PS microspheres being predominantly studied in terms of polymer and shape. As carriers for additives and environmental chemicals, inhalation exposure to MPs may pose additional risks to human health. Studies investigating the inhalation of MPs containing significant amounts of additives and/or pollutants are lacking. The transfer of MPs and endogenous/exogenous chemicals from the lungs into the bloodstream and/or lymphatic circulation still needs to be confirmed in humans, along with the local conditions favoring the release of these chemicals in the lungs. Similarly to other airborne particles, MPs may cross pulmonary membranes and accumulate in lymphatic nodules, impairing the immune system or being bioavailable for blood transport within the human body [74]. So far, only one article has reported the presence of MPs in human blood [68], leaving a significant gap in the knowledge on the target organs and Trojan Horse potential of these particles. Characterizing human exposure to MPs, monitoring plastic additives in the urine and blood of individuals with high exposure levels (e.g., workers in plastic industries and plastic recycling), and performing inhalation exposure studies using labeled MPs are promising research avenues for understanding the human health risk of MPs.

Chronic inhalation exposure to MPs, from birth to death, requires thorough investigations. Given the increasing prevalence of MPs in the air, a “One Health” approach integrating disciplines such as toxicology, environmental sciences, chemistry, biology, and medicine appears imperative. Only a multidisciplinary approach will enable a comprehensive understanding of the toxicological effects of MPs via inhalation, providing guidance for public health policies. Continuing the effort to characterize airborne MPs and those deposited in the respiratory system is important, considering all particle shapes, polymers, and even overlooked sources like rubber from tires. A non-target screening analysis is likely valuable for analyzing and studying the different chemicals associated with MPs in both human and environment samples, such as indoor buildings, urban centers, and plastic recycling areas. Nowadays, advanced instrumental methods are available for analyzing air and human biological samples. Encouraging the adoption of standardized protocols is important for consistently quantifying and characterizing MPs, considering factors like polymer types, sizes, shapes, and surface characteristics. Obtaining human samples poses ethical challenges, making them valuable and scarce resources in scientific research. Therefore, adopting a uniform approach can enhance the comparability of results across different studies, aiding in a comprehensive understanding of the health risks posed by airborne MPs.

## Figures and Tables

**Table 1:** MP characteristics in human lung tissues and bronchoalveolar lavage (BALF). The most abundant MP is highlighted in bold when reported by the studies. The size refers to either the average or the maximal size or the most abundant MP size found in the studies. Acrylates (ACRs), acrylic (AC), butadiene rubber (BR), chlorinated polyethylene (CPE), chlorinated polyisoprene (PICH), ethylenevinyl acetate (EVA), poly(ethylene terephthalate) (PET), poly(methylmethacrylate) (PMMA), poly(tetrafluoroethylene) (PTFE), poly-(vinylchloride) (PVC), polycarbonate (PC), polyester (PES), polyethylene (PE), polyimide (PI), polystyrene (PS), polysulfone (PSU), polytetrafluoroethylene (PTFE), polyurethane (PUR), polyamide (PA), polybutylene terephthalate (PBT), and polypropylene (PP).

Sample Type	Polymer	Shape	Size (μm)	Concentration	Detection Method	Reference
Lung tissue	**PP**, PET	Fibers, fragments, films	12–2475 (length) 4–88 (width)	0.41–3.12 MPs/g tissue	mFTIR	[2]
Lung tissue	**PP**, PET, PS, PVC, PTFE, CPE, PE, ACR, EVA, BR, PUR, silicone	Fibers	20–100 (diameter)	2.19 MPs/g tissue	LDIR	[49]
Lung tissue	**PP**, PE, PVC, cellulose acetate, PE, co-PP, PS, PS-co-PVC, PUR	Fibers, fragments	<5 (fragments) 8.12–16.8 (fibers)	0.56 MPs/g tissue	mRaman	[5]
Lung tissue	**PE**, PET, AC, phenoxy resin, rayon	Fibers (>20 μm)	Up to 1750 (length) Up to 34.29 (width)	-	mFTIR	[4]
BALF	-	Fibers	1730	9.2 ± 2.5 MPs/100 mL BALF	mFTIR	[3]
BALF	PP, PE, PES, PET, PVC, PC, PTFE, AC, PA, PBT	Fragment, fibers, pellets, sheet	<5	4.31 ± 2.77 MPs/10 mL BALF	mRaman	[50]
BALF	**PE**, PET, PP, PS, PC, PUR, PSU, PP, PVC, PMMA, PI, PTFE, ACR, PICH	Fiber (length-to-diameter ratio ≥3 μm), “Irregular particles” (length-to-diameter ratio <3 μm)	30–34	0.91 MPs/g BALF	LDIR	[6]

## Data Availability

Data are contained within the article.
